# Genetic variability in COVID-19-related genes in the Brazilian population

**DOI:** 10.1038/s41439-021-00146-w

**Published:** 2021-04-02

**Authors:** Rodrigo Secolin, Tânia K. de Araujo, Marina C. Gonsales, Cristiane S. Rocha, Michel Naslavsky, Luiz De Marco, Maria A. C. Bicalho, Vinicius L. Vazquez, Mayana Zatz, Wilson A. Silva, Iscia Lopes-Cendes

**Affiliations:** 1 Department of Translational Medicine, University of Campinas (UNICAMP), and The Brazilian Institute of Neuroscience and Neurotechnology (BRAINN), Campinas, SP Brazil; 2 Departament of Genetics and Evolutive Biology, Institute of Bioscience, University of São Paulo, (USP) and The Human Genome and Stem Cell Research Center, São Paulo, SP Brazil; 3 Department of Surgery, Federal University of Minas Gerais (UFMG), Belo Horizonte, MG Brazil; 4 Department of Clinical Medicine, Federal University of Minas Gerais (UFMG), Belo Horizonte, MG Brazil; 5 Molecular Oncology Research Center (CPOM), Barretos Cancer Hospital, Barretos, SP Brazil; 6 Department of Genetics, Ribeirão Preto Medical School, University of São Paulo at Ribeirão Preto (USP), Ribeirão Preto, SP Brazil

**Keywords:** Viral infection, Rare variants

## Abstract

SARS-CoV-2 utilizes the angiotensin-converting enzyme 2 (ACE2) receptor and transmembrane serine protease (TMPRSS2) to infect human lung cells. Previous studies have suggested that different host *ACE2* and *TMPRSS2* genetic backgrounds might contribute to differences in the rate of SARS-CoV-2 infection or COVID-19 severity. Recent studies have also shown that variants in 15 genes related to type I interferon immunity to influenza virus might predispose patients toward life-threatening COVID-19 pneumonia. Other genes (*SLC6A20, LZTFL1, CCR9, FYCO1, CXCR6, XCR1, IL6, CTSL*, *ABO*, and *FURIN*) and *HLA* alleles have also been implicated in the response to infection with SARS-CoV-2. Currently, Brazil has recorded the third-highest number of COVID-19 cases worldwide. We aimed to investigate the genetic variation present in COVID-19-related genes in the Brazilian population. We analyzed 27 candidate genes and *HLA* alleles in 954 admixed Brazilian exomes. We used the information available in two public databases (http://www.bipmed.org and http://abraom.ib.usp.br/) and additional exomes from individuals born in southeast Brazil, the region of the country with the highest number of COVID-19 patients. Variant allele frequencies were compared with the 1000 Genomes Project phase 3 (1KGP) and gnomAD databases. We detected 395 nonsynonymous variants; of these, 325 were also found in the 1KGP and/or gnomAD. Six of these variants were previously reported to influence the rate of infection or clinical prognosis of COVID-19. The remaining 70 variants were identified exclusively in the Brazilian sample, with a mean allele frequency of 0.0025. In silico analysis revealed that seven of these variants are predicted to affect protein function. Furthermore, we identified *HLA* alleles previously associated with the COVID-19 response at loci *DQB1* and *DRB1*. Our results showed genetic variability common to other populations and rare and ultrarare variants exclusively found in the Brazilian population. These findings might lead to differences in the rate of infection or response to infection by SARS-CoV-2 and should be further investigated in patients with this disease.

## COVID-19: Variants related to susceptibility in a Brazilian population

Genetic variants in the Brazilian population do not appear to explain the relatively high overall rates of COVID-19 cases compared to other countries, but could be relevant for risk assessment at the individual level. Iscia Lopes-Cendes of the University of Campinas and colleagues in Brazil examined gene sequences for variations in 27 genes reported to influence COVID-19 infection in Brazilians of mixed heritage and of people born in the southeast where case numbers are high. These studies included the *ACE2* gene that codes for a receptor allowing SARS-CoV-2 to enter host cells. Genetic variants were also identified in two international genome databases. Seventy variants were unique to Brazilians, but computer modeling predicted only seven variants that could affect protein function. The team also looked for and found variants that could affect individual immunity to COVID-19. Similar genetic studies could help identify at-risk individuals and therapeutic targets.

## Introduction

COVID-19 disease, caused by the coronavirus SARS-CoV-2, is currently a worldwide pandemic. SARS-CoV-2 used its spike protein to enter human lung cells, a process primed by the host serine protease TMPRSS2, followed by angiotensin-converting enzyme 2 (ACE2) receptor binding and proteolysis, with activation of membrane fusion within endosomes by cathepsin L (CTSL)^1–4^. The main feature of SARS-CoV-2 infection is preactivation of the spike protein by FURIN inside the host cell, which leads to increased SARS-CoV-2 spread into lung cells and increased virulence^5^. Rapid SARS-CoV-2 infection leads to an exacerbated immune reaction, and a few studies have shown that increased levels of IL-6 (an essential immune response mediator) are associated with an increased inflammatory response, respiratory failure, increased probability of intubation, the presence of clinical complications, and higher mortality in patients with COVID-19^6–8^. Additional studies have found enrichment of rare variants predicted to be loss-of-function mutations in genes related to type I interferon (IFN) immunity to influenza virus (*TLR3*, *TICAM1*, *TRIF*, *UNC93B1*, *TRAF3*, *TBK1*, *IRF3*, *NEMO*, *IKBKG*, *IFNAR1*, *IFNAR2*, *STAT1*, *STAT2*, *IRF7*, and *IRF9*) among patients with life-threatening COVID-19 pneumonia^9^.

Moreover, specific variants in the genes *ACE2* and *TMPRSS2* have been reported among diverse populations worldwide, suggesting that different host genetic backgrounds might contribute to differences in COVID-19 infection and severity^2,10^. Ellinghaus et al.^11^ performed a genome-wide association study (GWAS) including Italian and Spanish patients with confirmed COVID-19 and controls and identified six candidate genes associated with the COVID-19 response on chromosome (chr) 3p21.31 (*SLC6A20*, *LZTFL1*, *FYCO1*, *CXCR6*, *XCR1*, *CCR9*) and one on chr 9q34.2, the locus harboring genes encoding ABO blood group antigens. A subsequent, more extensive study replicated the association between the locus on chr 3p21.31 and COVID-19. This work revealed a core haplotype ranging from 45,859,651 bp to 45,909,024 bp that was inherited from Neanderthals and is currently carried by ~50% of people in South Asia and ~16% of people in Europe^12^. Interestingly, no evidence of association was found for the previously identified candidate genes potentially involved in the response to infection by SARS-CoV-2, namely, *ACE2*, *TMPRSS2*, *FURIN*, and *IL6*.

Furthermore, one significant factor modulating resistance or susceptibility to viral infections is the human leukocyte antigen (HLA) system. *HLA* polymorphism results from a set of amino acid substitutions in the peptide-binding groove of HLA molecules, producing variability in the peptide epitope-binding site and presentation to T cells, which may protect against epidemic infection^13^. Thus, genetic variability in *HLA* alleles might influence the immune response in patients with COVID-19, thereby modulating disease severity. Indeed, in silico analysis has found that HLA-B*46:01 has the fewest predicted binding sites for SARS-CoV-2 peptides and that HLA-B*15:03 shows the greatest capacity to present highly conserved shared SARS-CoV-2 peptides to immune cells^14^.

Brazil has reported the third-highest number of COVID-19 infections worldwide (updated on September 28th, 2020; https://covid19.who.int/; https://coronavirus.jhu.edu/map.html), and the highest number of cases is concentrated in the southeastern region of the country (updated on September 28th, 2020; https://covid.saude.gov.br/). A feature of Brazilian individuals is an admixed genome, encompassing European, sub-Saharan African, and Native-Americans as the three main ancestry populations^15–17^, and the distribution of ancestry components varies remarkably throughout the genome^18^. Furthermore, it has been demonstrated that a significant proportion of genetic variability in admixed Brazilians remains undiscovered^19^ and that genetic variability may lead to a differential response to infection^20^. Therefore, we aimed to investigate the genetic variation present in COVID-19-related genes in the Brazilian population.

## Results

### Exome analysis

We found 7172 variants among the candidate genes analyzed (Supplementary Table 1). Of these, 395 variants putatively impact protein function, including 354 nonsynonymous variants, seven frameshift substitutions, three in-frame deletions, one in-frame insertion, 12 stop-gain mutations, two start-loss mutations, and 16 splice site mutations (Supplementary Table 1). Three-hundred and twenty-five of the variants are present in the gnomAD and/or 1000 Genome Project phase 3 (1KGP) databases, including 56 common variants, with an alternative allele frequency (AAF) ≥ 0.01, and 269 rare variants (AAF < 0.01) (Supplementary Data 1). Although the AAF of the admixed Brazilian sample follows the distribution of non-Finish Europeans (NFE)/Europeans (EUR), sub-Saharan Africans (AFR), and admixed Americans (AMR) in the gnomAD and 1KGP databases (Supplementary Table 2), we observed differences in the AAF of these common and rare variants in the admixed Brazilian sample compared to the gnomAD^21^ and/or 1KGP^22^ databases (Supplementary Table 2 and Supplementary Data 1). Interestingly, we also detected some variability in the AAF among samples from different Brazilian towns and the two public databases of genomic information on the Brazilian population: BIPMed^19^ (www.bipmed.org) and ABraOM^23^ (http://abraom.ib.usp.br/) (Supplementary Table 3).

More importantly, 70 variants are exclusive to the Brazilian sample, including 11 variants in genes related to type I interferon (INF) immunity to influenza virus^9^, six in candidate genes for COVID-19 response identified by GWAS^11^, and five related to SARS-CoV-2 entry into lung cells and virus replication^2,10^. These variants are ultrarare, presenting a mean AF of 0.0025 (Supplementary Data 1). Among them, we found one in the dataset from Belo Horizonte and two in the ABraOM database, involving *ACE2* p.Arg219Cys, and one in the dataset from Barretos and two in the ABraOM database, involving *ACE2* p.Leu731Phe; the *TMPRSS2* p.Val160Met variant is present in samples from all different Brazilian towns and the two public databases (BIPMed and ABraOM), with an AAF ranging from 0.1333 in Belo Horizonte to 0.2931 in Campinas. Among the reported variants in genes influencing type I INF immunity to influenza virus^9^, we found three variants in the ABraOM database (*TLR3* p.Pro554Ser, *IFR3* p.Asn146Lys and *IRF7* p.Pro246Ser) (Supplementary Data1 and 2).

In addition, we identified five variants (rs35044562, rs34326463, rs35508621, rs67959919, and rs35624553), previously described by Zeberg & Pääbo^12^. These were only found in the samples from Ribeirão Preto and the BIPMed dataset (rs34326463), Campinas (rs35044562, and rs35508621), and the ABraOM dataset (rs35044562, rs35508621, rs67959919, and rs35624553) (Table 1).

**Table 1:** Alternative allele frequency (AAF) of variants encompassing the haplotype described by Zeberg and Pääbo^12^ and the alleles present in Neanderthal samples.

dbSNP	Chr	Pos	ALT	Brazil AF	Allele^a^	Campinas	Barretos	Ribeirão Preto	Belo Horizonte	BIPMed	ABraOM
rs35044562	3	45909024	G	0.0011	G	0.0862	0.0000	0.0000	0.0000	0.0279	0.0311
rs34326463	3	45899651	G	0.0011	G	0.0000	0.0000	0.0357	0.0000	0.0000	0.0000
rs35508621	3	45880481	C	0.0011	C	0.0345	0.0000	0.0000	0.0000	0.0000	0.0039
rs67959919	3	45871908	A	0.0011	A	0.0000	0.0000	0.0000	0.0000	0.0000	0.0039
rs35624553	3	45867440	G	0.0262	G	0.0000	0.0000	0.0000	0.0000	0.0000	0.0039

^a^Data extracted from Zeberg and Pääbo^12^.

Positions are based on the GRCh37 assembly.

### In silico predictions

We identified seven variants predicted to affect protein function in the 12 algorithms used: p.Phe249Ser, p.Gly164Val, and p.Leu25Pro in *SLC6A20*; p.Leu96Arg in *LZTFL1*; p.Tyr287Ser in *XCR1*; and p.Gly146Ser and p.Asn414Ser in *FURIN* (Table 2). The variant p.Gly146Ser in the *FURIN* gene is also predicted to destabilize the protein (ΔΔG: −1.576 kcal/mol). The p.Phe249Ser variant is present in samples from Barretos, the BIPMed dataset, gnomAD, and 1KGP (NFE/EUR, AFR, AMR, and SAS populations). In contrast, the p.Gly164Val variant is present in the ABraOM dataset, gnomAD, and 1KGP (NFE/EUR populations) and the p.Gly146Ser variant in the ABraOM dataset, gnomAD, and 1KGP (NFE/EUR, AFR, AMR, EAS, and SAS populations). Notably, four of the variants predicted to be deleterious are found exclusively in admixed Brazilian individuals (p.Leu25Pro in Barretos; p.Leu96Arg in the ABraOM dataset; p.Tyr287Ser in Belo Horizonte; and p.Asn414Ser in the BIPMed dataset). Also, we found the variant p.Pro554Ser in the *TLR3* gene, which is predicted as deleterious. However, the variant in the *IFR3* gene, (p.Asn146Lys) and in the *IRF7* gene (p.Pro246Ser) were not predicted to be deleterious according to our analysis (Supplementary Data 2).

**Table 2:** Alternative allele frequency of the deleterious variants found in the four COVID-related genes.

		Alternative allele frequency										
Genes	Variant	Campinas	Barretos	Ribeirão Preto	Belo Horizonte	ABraOM	BIPMed	NFE	AFR	AMR	EAS	SAS
*SLC6A20*	p.Phe249Ser	0	0.0167	0	0	0.0008	0.0019	0.0005	0.0615	0.0003	0	0.0084
*SLC6A20*	p.Gly164Val	0	0	0	0	0.0008	0	0.0088	0	0	0	0
*SLC6A20*	p.Leu25Pro	0	0.0167	0	0	0	0	0	0	0	0	0
*LZTFL1*	p.Leu96Arg	0	0	0	0	0.0008	0	0	0	0	0	0
*XCR1*	p.Tyr287Ser	0	0	0	0.0167	0	0	0	0	0	0	0
*FURIN*	p.Gly146Ser	0	0	0	0	0.0008	0	0.0004	0.0002	0.0005	0.0544	0.0327
*FURIN*	p.Asn414Ser	0	0	0	0	0	0.0019	0	0	0	0	0

*NFE* non-Finish European, *AFR* sub-Saharan African/African-American, *AMR* admixed Americans/Latinos, *EAS* east Asians, *SAS* south Asians.

We did not observe any predicted deleterious variants in *ACE2* and *TMPRSS2* based on our 12 algorithm criteria. However, Hou et al.^2^ applied only Polyphen2 and CADD scores to variants in *ACE2* and *TMPRSS2* (Polyphen2 > 0.96 and CADD > 20 as the cutoff). Therefore, only variants defined as “probably damaging” by Polyphen2 (http://genetics.bwh.harvard.edu/pph2/dokuwiki/overview) and CADD ( > 20) were included. We found 79 variants predicted to affect protein function, including the p.Val160Met variant in *TMPRSS2* reported by Hou et al.^2^ and the p.Pro554Ser variant in *TLR3* previously reported by Zhang et al.^9^ (Supplementary Data 2).

### HLA analysis

Overall, we identified 331 different *HLA* alleles in the admixed Brazilian samples. Of these, three *HLA* alleles have been previously associated with the COVID-19 response^14,24^. We compared the frequency of these *HLA* alleles in admixed Brazilians and in populations that occupy the top 10 positions for most cases of COVID-19 and the five populations less affected by the disease, including the United States, India, Russia, Colombia, Peru, Mexico, Spain, Argentina, South Africa, Japan, Australia, South Korea, Vietnam, and Taiwan. The frequency of these alleles is described in Supplementary Data 3. The HLA-B*46:01, HLA-B*27:07, HLA-B*15:27, and HLA-C*07:29 alleles are absent in the Brazilian samples. The HLA-C*07:29 allele is also absent from other populations and is present at a low frequency (AF = 0.0003) in the Indian population. HLA-B*15:27 was identified in Vietnam, Taiwan, Japan, with an AF > 0.01, and Spain, with an AF < 0.0001. HLA-B*27:07 was detected at a low frequency in India, Colombia, Spain, and South Africa; HLA-B*46:01 allele is present in Russia, Mexico, Vietnam, Taiwan, and Japan.

Sixty-six Brazilian individuals (17.1%) carry the HLA-DQB1*06:02 allele (AF = 0.08938) and 47 individuals (12.2%) the HLA-DRB1*15:01 allele (AF = 0.06477); 32 individuals (8.29%) harbor both the HLA-DRB1*15:01 and HLA-DQB1*06:02 alleles. The populations of continents other than Oceania also have these two *HLA* alleles (HLA-DRB1*15:01 and HLA-DQB1*06:02), with an AF > 0.01. Additionally, 15 Brazilian individuals (3.88%) carry HLA-B*15:03 (AF = 0.02073), which is predicted to have the greatest capacity to present SARS-CoV-2 peptides to immune cells^14^. This allele was not found in the Asian population of Japan, South Korea, or Vietnam (Supplementary Data 3).

### In silico analysis of viral peptide-HLA class I- and II- binding affinity

To verify the potential for an *HLA* allele type to produce an antiviral response to SARS-CoV-2, we performed *HLA*- binding affinity analyses on the SARS-CoV-2 proteome. We tested 42 HLA-A, 77 HLA-B, 38 HLA-C, 60 HLA-DP (DPA1/DPB1), 145 HLA-DQ (DQA1/DQB1), 46 HLA-DRB1, 4 DRB3, 2 DRB4, and 6 DRB5 proteins.

The SARS-CoV-2 proteome exhibits a diversity of *HLA* alleles from classes I and II (Supplementary Table 4). HLA proteins are predicted to bind a small proportion of all possible SARS-CoV-2-derived peptides with high affinity (on average 0.5% for HLA class I and 2% for HLA class II). Additionally, we found a small proportion of weak binders (on average, 1.5% for HLA class I and 8.2% for class II). Most of the HLA proteins do not bind either class I (on average >96%) or II (on average >89%) molecules (Supplementary Data 4). Supplementary Data 5 shows a list of the strongest HLA binders (>300 peptides bound at high affinity) of SARS-CoV-2 peptides. These are found at loci *HLA-A*, *-B*, *-C*, and *-DQ*. When comparing HLA class I and class II, HLA molecules do not share similar characteristics or partial amino acid sequences.

## Discussion

Accessing the genomic sequences of the general population is relevant for identifying the genetic variability involved in the molecular mechanisms of infection^20^. Additionally, it is known that the admixed Brazilian population is underrepresented in large public databases^21,22^, and previous studies have revealed variants exclusively present in Brazilian individuals^19,23^. Thus, we hypothesize that rare or ultrarare variants of large effect sizes might make an essential contribution to COVID-19 infection in the Brazilian population.

We studied 27 human COVID-19-related genes and the *HLA* region in two public genomic databases of admixed Brazilians (BIPMed and ABraOM) and additional samples from individuals born in three different towns of southeastern Brazil. We examined the variants and *HLA* alleles found in these samples and compared them with worldwide populations. We also report variants constituting a haplotype at locus 3p21.31 that is described as being inherited from Neanderthals^12^.

Previous studies have shown that the *ACE2*, *TMPRSS2*, *CTSL*, *FURIN*, and *IL6* genes, as well as the *HLA* region, may be involved in SARS-CoV-2 infection^1–5,10^ and the immune response^6–8,14,24,25^. Furthermore, variants at loci 3p21.31 and 9q34.2 (encompassing *SLC6A20*, *LZTFL1*, *FYCO1*, *CXCR6*, *XCR1*, *CCR9*, and *ABO*) have been associated with Spanish and Italian patients with COVID-19^11^, and different variants affect predisposition toward life-threatening illness in COVID-19 patients of different ancestries^9^.

Overall, analysis of genetic variability in candidate genes for specific populations can help identify individuals at a higher risk of infection or severe disease by constructing risk haplotypes, which may also provide therapeutic targets for the development of more effective treatments and the control of COVID-19^2,10^. Thus, in addition to investigating genetic variability in 27 candidate genes, we extended our analysis to include *HLA* alleles, which influence the immunological response to many infectious agents^26^. We report the first comprehensive study of the genetic variability of genes related to COVID-19 in admixed individuals from Latin America, a population strongly affected by the COVID-19 pandemic, both in terms of the number of infected individuals and the severity of disease, leading to increased death rates (updated on September 28th, 2020; https://covid19.who.int/; https://coronavirus.jhu.edu/map.html). Indeed, in the USA, remarkable disparities in SARS-CoV-2 infection based on ethnicity have been shown, with Hispanic/Latino and African-American individuals presenting higher SARS-CoV-2 infection rates and risk mortality than “non-Hispanic white” Americans^27–29^. Therefore, by examining population genomics data, one may gain insight into disease-related variants, which may be disproportionally represented in specific populations^18,30–32^. Furthermore, by evaluating individuals with unknown information regarding SARS-CoV-2 infection, one can achieve the random distribution of these variants, allowing for better estimates of the distribution of population allele frequencies.

The spread of COVID-19 infection displays geographical variation. Most COVID-19-related deaths are being reported in the Americas, Europe, and Southeast Asia, with fewer deaths in Oceania and East Asia. Considering the ratio of deaths to the total number of cases, Mexico has the highest proportion, at 10%, whereas the USA, Brazil, Colombia, Australia, and Vietnam have a proportion of ~3% (“https://www.worldometers.info/coronavirus/”; accessed on 04/24/2020). The reasons for this geographical variation are still unclear.

We identified small AF differences in the 395 candidate variants identified among Brazilian samples, strengthening the hypothesis that different genetic backgrounds might influence SARS-CoV-2 infection and behavior in human host cells^2,10^. Moreover, this study and previous works^2,10^ identified individuals who carry unique deleterious variants, which may influence gene function and potentially lead to different responses to SARS-CoV-2 infection on an individual scale. Nevertheless, the rather similar distribution of AFs among Brazilians and their ancestry populations (NFE/EUR and AFR), as well as other admixed Americans (AMR), and the fact that the unique variants identified in the Brazilian population are rare or ultrarare indicate that the admixed Brazilian genetic background is not sufficient to influence SARS-CoV-2 infection on a population scale. However, we cannot exclude the possibility that some of the rare and ultrarare variants identified in Brazilian individuals in this study may affect disease risk at the individual level. In general, predisposition toward infectious diseases is most likely multifactorial, with genetic and environmental influences^20,33^. When examining the genetic component of susceptibility to infection, the literature highlights polygenic inheritance; indeed, several variants have been associated with infectious disease, each leading to small increments of disease risk^20,33^. In this context, rare variants may also play a relevant role in infectious diseases, such as in HIV-1^33^, HSV-1^34^, and invasive meningococcal disease^35^, as well as reported previously for COVID-19^36^.

Zeberg and Pääbo^12^ have shown that a major genetic risk factor for severe COVID-19 may be inherited from Neanderthals^12^. This finding is important on a regional scale, as 4% of the admixed Americans analyzed by Zeberg and Pääbo^12^ (including 1533 Brazilian controls from the BRACOVID dataset) presented the core haplotype derived from Neanderthals. Interestingly, data from Campinas, Ribeirão Preto, and the BIPMed dataset showed only one allele, and Barretos and Belo Horizonte did not present any allele deriving from Neanderthals in the core haplotype reported. Therefore, if further studies demonstrate that the Neanderthal-derived locus indeed confers a genetic risk to COVID-19, this information should be carefully evaluated in additional admixed Brazilian samples from different geographic areas.

Currently, there is no consensus regarding a possible association of *HLA* alleles and susceptibility to COVID-19. For example, Ellinghaus et al.^11^ did not find any evidence of an association between *HLA* and COVID-19. Conversely, HLA-DRB1*15:01, HLA-DQB1*06:02, and HLA-B*27:07 alleles have been associated with Italian cases of an extremely severe or severe form of COVID-19^24^, and an increased frequency of HLA-C*07:29 and HLA-B*15:27 was detected in Chinese patients with COVID-19 in comparison to the Chinese control population^25^. Interestingly, the HLA-C*07:29 allele was absent from the Brazilian admixed samples included in the present study and in all populations used in the comparisons, except for individuals from India, where this allele occurs at a low frequency (0.0003). On the other hand, the HLA-B*15:27 allele was identified in individuals from three Asian countries (Vietnam, Taiwan, and Japan), with an AF > 0.01, and at a low frequency in Spain (0.0001) but is absent in Brazilian samples. The HLA-B*27:07 allele was detected in Italian individuals with a severe manifestation of COVID-19 and also identified in India, Colombia, Spain, and South Africa but not in populations from Asia and Oceania (countries that are less affected by COVID-19) and Brazil. In contrast, HLA-DQB1*06:02 is present in all populations surveyed in this study, including Brazilian individuals (17.1%), with the exception of individuals from Australia. The HLA-DRB1*15:01 allele is also present in all populations investigated in this study, including Brazilian individuals (12.2%), but not in individuals from Australia and Peru. Interestingly, 8.29% of Brazilian individuals carry both the HLA-DRB1*15:01 and HLA-DQB1*06:02 alleles. It is important to point out that our study has a significant advantage over previous reports about the study of HLA in the Brazilian population, as we performed NGS-based HLA typing, which has a high resolution^37–39^.

The *HLA-A*, *-B*, -C, and *-DQ* loci present haplotypes in the Brazilian samples that encode strong binders of SARS-CoV-2 peptides, especially for the *HLA-A* locus (20 alleles, Table 3). One hypothesis is that those who express strong HLA binders, i.e., those in which HLA sites would be more efficient at binding SARS-CoV-2 peptides and presenting it to the immune system, would be less susceptible to infection or would develop only mild disease. In our analysis, this low-risk category, strongest binders, included individuals caring *HLA* alleles predicted to encode proteins binding >300 peptides of the SARS-CoV-2 at high affinity. Thus, considering sites on *HLA A*, -*B*, -*C*, and -*DQA1-DQB1*, the proportion of strongest binders in the Brazilian population may be up to 51% (198 individuals), and some individuals carry alleles for the strongest binders at more than one site (Table 3). Accordingly, we firmly believe that this issue should be further investigated in large cohorts of patients with COVID-19 in the Brazilian population.

**Table 3:** List of the strongest HLA binders (>300 peptides bound at high affinity) of SARS-CoV-2 peptides and frequency in the Brazilian sample.

*HLA*	*HLA* alleles	Allele frequency
*HLA-A*	A*01:01	0.10233
	A*11:01	0.04145
	A*11:67	0.00130
	A*23:01	0.03756
	A*23:17	0.00389
	A*24:02	0.10104
	A*24:03	0.00259
	A*24:05	0.00130
	A*25:01	0.00389
	A*26:01	0.02979
	A*26:02	0.00130
	A*26:08	0.00130
	A*29:01	0.00259
	A*29:02	0.04534
	A*29:119	0.00130
	A*30:02	0.02591
	A*30:04	0.00259
	A*34:02	0.00777
	A*36:01	0.00389
	A*80:01	0.00130
*HLA-B*	B*15:08	0.00130
	B*15:11	0.00130
*HLA-C*	C*03:02	0.00389
	C*07:02	0.05699
	C*07:50	0.00130
	C*14:02	0.02979
	C*14:03	0.00259
*HLA-DQ*	DQA1*02:01-DQB1*04:02	0.00259
	DQA1*03:01-DQB1*04:02	0.00130
	DQA1*03:03-DQB1*04:01	0.00130
	DQA1*03:03-DQB1*04:02	0.00130

When comparing different populations, we found marked variability in the frequency of the different *HLA* alleles putatively associated with severe manifestation of COVID-19, such as HLA-DRB1*15:01, HLA-DQB1*06:02, and HLA-B*27:07 alleles^23^. Overall, 10% of Brazilian individuals carry at least two of the alleles associated with severe manifestation of COVID-19. Interestingly, the same alleles are absent in individuals from Australia. Variability in the frequency of *HLA* alleles previously associated with COVID-19 highlights the importance of considering ethnic and geographic origin when performing studies investigating the role of *HLA* alleles and disease. Thus, it seems likely that different population-specific haplotypes may be associated with an increased genetic risk of the disease in different populations.

In conclusion, we detected three rare and four ultrarare variants in four COVID-19-related genes, *SLC6A20, LZTFL1, XCR1* and *FURIN*, that are present only in the Brazilian dataset and predicted to affect protein function. Furthermore, we identified *HLA* alleles previously associated with the COVID-19 immunological response and 31 *HLA* alleles predicted to encode strong binders to SARS-CoV-2 peptides at loci *-A*, *-B*, *-C*, and *-DQ*, which indicates the importance of further investigation on the role of *HLA* haplotypes as modulators of the response to infection with SARS-CoV-2. Although variants of COVID-19-related genes predicted to affect protein function are rare in admixed Brazilians (varying from 0.0001 to 0.0032), they also emerge as candidates for modulating the response to infection by SARS-CoV-2 in the Brazilian population. Furthermore, our study suggests the utility of population genomic studies in the context of precision health to stratify risk of infections.

## Methods

### Subjects

We evaluated exomes of 257 individuals from the BIPMed dataset^19^ and 609 from the ABraOM dataset^23^ and an additional 88 exomes from individuals born in three towns in southeastern Brazil: Barretos (*N* = 30), Ribeirão Preto (*N* = 30), located in the state of São Paulo, and Belo Horizonte (*N* = 28), the capital of the state of Minas Gerais. Among the BIPMed individuals, information about their city of birth was available for 193. The HLA region was sequenced in 386 individuals, including 257 from BIPMed, 88 additional exomes, and an additional 41 individuals (22 from southeast Brazil). We signed terms of data privacy to obtain permission to use the raw data from BIPMed and ABraOM public databases as well as the raw data of the 88 exomes from Barretos, Ribeirão Preto, and Belo Horizonte. This study was approved by the University of Campinas Research Ethics Committee (UNICAMP, Campinas, São Paulo, Brazil). All methods were performed according to the relevant guidelines and regulations.

### Exome analysis

Whole-exome data were stored in variant call format (VCF) files built-in GRCh37 assembly. Gene regions were extracted by *VCFtools*^40^ based on the position reported in Ensembl GRCh37 Release 101^41^ (Supplementary Table 1). Consequences of variants were annotated from each gene region by ANNOVAR software (version 2019Oct24)^42^ using the following flags: *-otherinfo* (to include Brazil AF); *-onetranscript*; *-buildver hg19*; *-remove*; *-protocol refGene, gnomad211_exome*; *ALL.sites.2015_08*; *EUR.sites.2015_08*; *AFR.sites.2015_08*; *AMR.sites.2015_08*; *EAS.sites.2015_08*; *SAS.sites.2015_08*; *-operation g,f*; and *-nastring*. ANNOVAR software provides allele frequency (AF) information for African/African-American (AFR/AFA), Latino/admixed American (LAT/AMR), East Asian (EAS), non-Finish European (NFE), and South Asian (SAS) populations from the gnomAD exome dataset^21^, as well as sub-Saharan Africans (AFR), Europeans (EUR), admixed Americans (AMR), East Asians (EAS), and South Asians (SAS) from the 1KGP phase 3 dataset^22^. In addition, we annotated variants that were not identified by ANNOVAR using the Variant Effect Prediction (VEP) algorithm^43^, with the following parameters: *--buffer_size 500*; *--canonical*; *--distance 5000*; *--species homo_sapiens*; *--symbol*. We defined rare variants as those with allele frequency < 0.01^22^; ultrarare variants are rare variants present in our data but absent in gnomAD and 1KGP^44–46^.

To evaluate regional variability among Brazilian samples, we separated the samples based on the city in which the individuals were born, including 32 individuals from Campinas extracted from the BIPMed dataset.

### In silico prediction analysis

To predict the impact on protein function of the nonsynonymous variants identified, we applied the following computer algorithms, which are currently recommended by the ACMG/AMP guidelines: PANTHER^47^, MutationTaster^48^, Condel^49^, PROVEAN^50^, PolyPhen2^51^, Sort Intolerant from tolerant (SIFT)^52^, Align Grantham Variation/Grantham Difference score (GVGD)^53^, Combined Annotation Dependent Depletion (CADD)^54^, PhD-SNPg^55^, Functional Analysis through Hidden Markov Models (FATHMM)^56^, SNPs&GO^57^, and MutPred2 (http://mutpred.mutdb.org).

For Align-GVGD, we classified the variants based on the graded classifier, with a cutoff of C35 or higher for deleterious classification. For CADD, we used the PHRED-like score with a cutoff of 20, below which variants were classified as benign and otherwise deleterious. For MutPred2, we considered a score threshold of 0.50 for pathogenicity. For all other algorithms, we considered the classification provided as output.

We used the DynaMut server (http://biosig.unimelb.edu.au/dynamut/) to assess the impact of mutations on protein dynamics and stability^58^. The server requires an input file of protein structure in PDB format or by providing the four-letter accession code for any entry at the Protein Data Bank database (PDB; http://wwpdb.org). The used code for the *FURIN* gene was 5jxg. The other proteins are not available in PDB.

### HLA analysis

We sequenced 11 *HLA* loci (*HLA-A, HLA-B, HLA-C, HLA-DRB1, HLA-DQB1, HLA-DPB1, HLA-DQA1, HLA-DPA1, HLA-DRB3, HLA-DRB4, HLA-DRB5*) in 298 samples using NGSgo^®^ panels (GenDx, Utrecht, The Netherlands). The DNA libraries were loaded onto a MiSeq Sequencer (Illumina Inc., San Diego, CA, USA), and the data were analyzed with NGSengine v.2.16.2 software (GenDx, Utrecht, The Netherlands). We determined *HLA* alleles from the remaining 88 exomes using the HLA-HD (HLA typing from High-quality Dictionary) tool v.1.3.0^37–39^. The IPD-IMGT/HLA database release 3.40.0^59^ was used as a reference. Although we obtained results with six- and eight-digit precision, we restricted the results to four-digit accuracy for comparison with published data. *HLA* allele frequencies were calculated by Arlequin v.3.5.2.2 software^60^.

### In silico analysis of viral peptide-HLA class I- and II- binding affinity

We performed in silico analysis of viral peptide-HLA class I- and II- binding affinity across HLA proteins found in our population for the entire SARS-CoV-2 proteome. All *HLA-A*, *-B*, and *-C* alleles were selected to assess the peptide‐binding affinity of their corresponding proteins HLA-A, HLA-B, and HLA-C, respectively. *HLA-DR* is represented by HLA-DRA/DRB1 dimer. Since HLA-DRA is considered monomorphic, we only used HLA-DRB1. *HLA-DP* and *HLA-DQ* are represented by the HLA-DPA1/DPB1 dimer and HLA-DQA1/DQB1 dimer, respectively.

FASTA-formatted protein sequence data were retrieved from the National Center of Biotechnology Information (NCBI) database (https://www.ncbi.nlm.nih.gov/genome/browse#!/overview/Sars-cov-2). The following eleven protein viral products were used in the analyses: ORF1ab (YP_009724389.1), surface glycoprotein (S) (YP_009724390.1), ORF3a (YP_009724391.1), envelope (E) (YP_009724392.1), membrane glycoprotein (M) (YP_009724393.1), ORF6 (YP_009724394.1), ORF7a (YP_009724395.1), ORF7b (YP_009725318.1), ORF8 (YP_009724396.1), nucleocapsid (N) (YP_009724397.2), and ORF10 (YP_009725255.1).

We k-merized these sequences into 8- to 12-mers to assess HLA class I-peptide-binding affinity and into 15-mers to assess HLA class II-binding affinity across the entire proteome. Predictions for HLA were performed using different *HLA* alleles found in our population with netMHCpan v4.1 for class I^61^ and NetMHCIIpan-3.2 for class II^62^. We followed the NetMHCpan recommendations, in which the information of strong MHC binder (SB) or weak MHC binder (WB) is based on a %Rank score. We used the default, %Rank < 0.5% and %Rank < 2% thresholds to detect SBs and WBs, respectively, for class I and %Rank < 2% and %Rank < 10% for SBs and WBs, respectively, for class II.

### HLA allele and haplotype frequencies of other populations

*HLA* frequency data were obtained from Allele Frequency Net Database (http://www.allelefrequencies.net/)^63^ for 10 distinct populations that are most and least affected by COVID-19. We checked the *HLA* of the populations that occupied the top 10 positions (USA, India, Brazil, Russia, Colombia, Peru, Spain, Mexico, Argentina, South Africa) and those that were less affected (Australia, Vietnam, Taiwan, Japan, and South Korea) (accessed on 04/24/2020, https://www.worldometers.info/coronavirus/) according to the availability of these data in Allele Frequency Net Database.

## Supplementary information

Supplementary Tables.

Supplementary data 1.

Supplementary data 2.

Supplementary data 3.

Supplementary data 4.

Supplementary data 5.

## Data Availability

The raw BIPMed dataset that supports this study’s findings is available in the EVA repository/PRJEB39251, https://www.ebi.ac.uk/eva/?eva-study=PRJEB39251. The ABraOM raw dataset that supports the results of this study is available from ABraOM (http://abraom.ib.usp.br/).
