# Supplementary material for: Genetic variability in COVID-19-related genes in the Brazilian population
Source: Hum Genome Var. 2021 Apr 2;8:15. doi: 10.1038/s41439-021-00146-w (PMC8017521; doi:10.1038/s41439-021-00146-w)
Supplement: Supplementary file 1 — Supplementary Tables. [file 41439_2021_146_MOESM1_ESM.docx]

**Supplementary information**

**Genetic variability in COVID-19-related genes in the Brazilian population**

Rodrigo Secolin, Marina C. Gonsales, Tania K. Araújo, Cristiane S. Rocha, Michel Naslavsky, Luiz De Marco, Maria A.C. Bicalho, Vinicius L. Vazquez, Mayana Zatz, Wilson A. Silva, Iscia Lopes-Cendes

Supplementary Table 1. Gene regions used to extract variants from the VCF files, including the number of variants and coding variants per candidate gene studied. Positions are based on GRCh37/hg19 assembly.

| **Gene** | **Chr** | **Start (bp)** | **End (bp)** | **Number of variants** | **Number of coding variants*** |
| --- | --- | --- | --- | --- | --- |
| *STAT1* | 2 | 191833762 | 191878976 | 411 | 6 |
| *SLC6A20* | 3 | 45796942 | 45838027 | 351 | 18 |
| *LZTFL1* | 3 | 45864808 | 45957534 | 447 | 7 |
| *CCR9†* | 3 | 45927996 | 45944667 | 131† | 5 |
| *FYCO1* | 3 | 45959396 | 46037316 | 681 | 67 |
| *CXCR6* | 3 | 45982425 | 45989845 | 44†† | 3 |
| *XCR1* | 3 | 46058516 | 46069234 | 88 | 2 |
| *TLR3* | 4 | 186990309 | 187006252 | 142 | 19 |
| *IL6* | 7 | 22765503 | 22771621 | 92 | 7 |
| *CTSL* | 9 | 90340434 | 90346308 | 99 | 11 |
| *ABO* | 9 | 136125788 | 136150617 | 493 | 31 |
| *IRF7* | 11 | 612555 | 615999 | 100 | 25 |
| *UNC93B1* | 11 | 67758575 | 67771593 | 201 | 21 |
| *STAT2* | 12 | 56735381 | 56754058 | 198 | 14 |
| *TBK1* | 12 | 64845840 | 64895899 | 355 | 16 |
| *IRF9* | 14 | 24630422 | 24635774 | 72 | 3 |
| *TRAF3* | 14 | 103243816 | 103377837 | 987 | 8 |
| *FURIN* | 15 | 91411822 | 91426688 | 236 | 23 |
| *TICAM1/TRIF* | 19 | 4815936 | 4831754 | 228 | 24 |
| *IRF3* | 19 | 50162826 | 50169132 | 98 | 22 |
| *IFNAR2* | 21 | 34602231 | 34636831 | 336 | 13 |
| *IFNAR1* | 21 | 34696748 | 34732129 | 273 | 12 |
| *TMPRSS2* | 21 | 42836478 | 42903043 | 765 | 21 |
| *ACE2* | X | 15579156 | 15620271 | 221 | 12 |
| *NEMO/IKBKG* | X | 153769419 | 153796804 | 123 | 5 |
| **Total** |  |  |  | **7172** | **395** |

**Notes**: *missense variants, frameshifts, stop gained, and splicing sites; ^†^overlap with *LZTFL1*; ^††^overlap with *FYCO1*; Chr= chromosome.

Supplementary Table 2. Distribution of alternative allele frequency of common variants in the candidate genes studied (AAF>0.01) found in the samples of admixed Brazilians and worldwide public datasets. Positions are based on GRCh37/hg19 assembly.

| **Gene** | **Chr** | **Start** | **Brazil** | **gnomAD** | | | | | **1KGP** | | | | |
| --- | --- | --- | --- | --- | --- | --- | --- | --- | --- | --- | --- | --- | --- |
|  |  |  |  | **NFE** | **AFR** | **AMR** | **EAS** | **SAS** | **EUR** | **AFR** | **AMR** | **EAS** | **SAS** |
| *SLC6A20* | 3 | 45814094 | 0.048 | 0.097 | 0.017 | 0.047 | 0.005 | 0.038 | 0.092 | 0.005 | 0.043 | 0.006 | 0.031 |
| *SLC6A20* | 3 | 45837886 | 0.147 | 0.159 | 0.027 | 0.074 | 0.055 | 0.133 | 0.158 | 0.011 | 0.112 | 0.083 | 0.110 |
| *LZTFL1* | 3 | 45869972 | 0.078 | 0.082 | 0.041 | 0.040 | 0.043 | 0.075 | 0.074 | 0.043 | 0.038 | 0.039 | 0.077 |
| *LZTFL1* | 3 | 45875738 | 0.014 | 0.000 | 0.071 | 0.002 | 0.000 | 0.000 | 0.000 | 0.071 | 0.007 | 0.000 | 0.000 |
| *FYCO1* | 3 | 45960562 | 0.014 | 0.000 | 0.000 | 0.000 | 0.000 | 0.000 | 0.426 | 0.424 | 0.533 | 0.674 | 0.375 |
| *CXCR6* | 3 | 45987980 | 0.101 | 0.002 | 0.446 | 0.021 | 0.001 | 0.002 | 0.005 | 0.491 | 0.068 | 0.000 | 0.001 |
| *FYCO1* | 3 | 46007823 | 0.042 | 0.111 | 0.017 | 0.061 | 0.003 | 0.339 | 0.122 | 0.005 | 0.059 | 0.004 | 0.360 |
| *FYCO1* | 3 | 46007825 | 0.050 | 0.113 | 0.029 | 0.065 | 0.003 | 0.340 | 0.123 | 0.019 | 0.063 | 0.004 | 0.359 |
| *FYCO1* | 3 | 46007846 | 0.042 | 0.012 | 0.156 | 0.034 | 0.000 | 0.003 | 0.015 | 0.174 | 0.040 | 0.000 | 0.001 |
| *FYCO1* | 3 | 46008647 | 0.012 | 0.000 | 0.052 | 0.004 | 0.000 | 0.033 | 0.004 | 0.063 | 0.004 | 0.000 | 0.000 |
| *FYCO1* | 3 | 46008790 | 0.397 | 0.424 | 0.132 | 0.606 | 0.669 | 0.359 | 0.431 | 0.093 | 0.565 | 0.661 | 0.372 |
| *FYCO1* | 3 | 46009487 | 0.075 | 0.112 | 0.028 | 0.061 | 0.003 | 0.340 | 0.122 | 0.017 | 0.059 | 0.005 | 0.359 |
| *FYCO1* | 3 | 46009864 | 0.495 | 0.538 | 0.236 | 0.677 | 0.677 | 0.699 | 0.556 | 0.210 | 0.643 | 0.667 | 0.731 |
| *FYCO1* | 3 | 46009981 | 0.015 | 0.001 | 0.061 | 0.005 | 0.000 | 0.000 | 0.001 | 0.082 | 0.012 | 0.000 | 0.000 |
| *FYCO1* | 3 | 46010077 | 0.868 | 0.776 | 0.956 | 0.925 | 1.000 | 0.901 | 0.801 | 0.982 | 0.914 | 1.000 | 0.922 |
| *TLR3* | 4 | 187004074 | 0.269 | 0.298 | 0.065 | 0.299 | 0.341 | 0.238 | 0.324 | 0.027 | 0.306 | 0.328 | 0.263 |
| *TLR3* | 4 | 187004767 | 0.013 | 0.000 | 0.032 | 0.002 | 0.000 | 0.000 | 0.001 | 0.040 | 0.007 | 0.000 | 0.000 |
| *IL6* | 7 | 22771039 | 0.020 | 0.009 | 0.004 | 0.144 | 0.026 | 0.008 | 0.017 | 0.002 | 0.078 | 0.026 | 0.003 |
| *ABO* | 9 | 136131289 | 0.275 | 0.237 | 0.246 | 0.490 | 0.264 | 0.207 | 0.221 | 0.243 | 0.431 | 0.290 | 0.220 |
| *ABO* | 9 | 136131315 | 0.079 | 0.078 | 0.168 | 0.055 | 0.182 | 0.260 | 0.085 | 0.169 | 0.048 | 0.194 | 0.233 |
| *ABO* | 9 | 136131316 | 0.023 | 0.021 | 0.004 | 0.011 | 0.000 | 0.008 | 0.029 | 0.003 | 0.017 | 0.000 | 0.008 |
| *ABO* | 9 | 136131322 | 0.079 | 0.077 | 0.167 | 0.055 | 0.182 | 0.259 | 0.085 | 0.169 | 0.048 | 0.194 | 0.233 |
| *ABO* | 9 | 136131415 | 0.080 | 0.077 | 0.168 | 0.057 | 0.183 | 0.260 | 0.085 | 0.169 | 0.048 | 0.194 | 0.233 |
| *ABO* | 9 | 136131472 | 0.274 | 0.221 | 0.233 | 0.467 | 0.244 | 0.198 | 0.221 | 0.242 | 0.431 | 0.289 | 0.220 |
| *ABO* | 9 | 136131523 | 0.015 | 0.023 | 0.005 | 0.008 | 0.000 | 0.002 | 0.025 | 0.005 | 0.014 | 0.000 | 0.001 |
| *ABO* | 9 | 136131576 | 0.024 | 0.017 | 0.005 | 0.131 | 0.000 | 0.033 | 0.012 | 0.001 | 0.108 | 0.000 | 0.026 |
| *ABO* | 9 | 136131592 | 0.100 | 0.099 | 0.171 | 0.066 | 0.182 | 0.267 | 0.114 | 0.174 | 0.065 | 0.194 | 0.241 |
| *ABO* | 9 | 136131651 | 0.099 | 0.071 | 0.225 | 0.038 | 0.175 | 0.034 | 0.099 | 0.256 | 0.068 | 0.151 | 0.029 |
| *ABO* | 9 | 136133506 | 0.730 | 0.797 | 0.734 | 0.515 | 0.734 | 0.791 | 0.805 | 0.725 | 0.576 | 0.711 | 0.770 |
| *ABO* | 9 | 136135238 | 0.445 | 0.760 | 0.681 | 0.508 | 0.733 | 0.793 | 0.778 | 0.674 | 0.569 | 0.710 | 0.774 |
| *ABO* | 9 | 136136770 | 0.713 | 0.759 | 0.685 | 0.512 | 0.734 | 0.792 | 0.794 | 0.688 | 0.578 | 0.710 | 0.775 |
| *ABO* | 9 | 136136773 | 0.023 | 0.018 | 0.036 | 0.009 | 0.000 | 0.002 | 0.020 | 0.039 | 0.017 | 0.000 | 0.001 |
| *ABO* | 9 | 136137547 | 0.021 | 0.021 | 0.004 | 0.011 | 0.000 | 0.007 | 0.029 | 0.004 | 0.017 | 0.000 | 0.008 |
| *IRF7* | 11 | 613208 | 0.345 | 0.263 | 0.513 | 0.320 | 0.028 | 0.140 | 0.267 | 0.585 | 0.303 | 0.021 | 0.109 |
| *IRF7* | 11 | 614318 | 0.346 | 0.266 | 0.524 | 0.323 | 0.028 | 0.140 | 0.267 | 0.585 | 0.303 | 0.021 | 0.109 |
| *UNC93B1* | 11 | 67759316 | 0.119 | 0.020 | 0.012 | 0.005 | 0.005 | 0.004 | 0.000 | 0.000 | 0.000 | 0.000 | 0.000 |
| *UNC93B1* | 11 | 67763282 | 0.013 | 0.019 | 0.003 | 0.006 | 0.000 | 0.016 | 0.020 | 0.000 | 0.004 | 0.000 | 0.013 |
| *UNC93B1* | 11 | 67765163 | 1.000 | 1.000 | 1.000 | 1.000 | 1.000 | 1.000 | 1.000 | 1.000 | 1.000 | 1.000 | 1.000 |
| *STAT2* | 12 | 56737251 | 0.012 | 0.010 | 0.001 | 0.005 | 0.000 | 0.002 | 0.008 | 0.000 | 0.006 | 0.000 | 0.003 |
| *STAT2* | 12 | 56740682 | 0.045 | 0.064 | 0.009 | 0.054 | 0.029 | 0.024 | 0.067 | 0.002 | 0.056 | 0.036 | 0.019 |
| *STAT2* | 12 | 56742997 | 0.047 | 0.001 | 0.204 | 0.016 | 0.000 | 0.000 | 0.001 | 0.224 | 0.025 | 0.000 | 0.000 |
| *STAT2* | 12 | 56743044 | 0.026 | 0.000 | 0.001 | 0.112 | 0.009 | 0.000 | 0.000 | 0.000 | 0.105 | 0.004 | 0.002 |
| *TRAF3* | 14 | 103342049 | 0.405 | 0.231 | 0.835 | 0.448 | 0.402 | 0.172 | 0.248 | 0.906 | 0.486 | 0.398 | 0.172 |
| *FURIN* | 15 | 91419098 | 0.025 | 0.003 | 0.077 | 0.008 | 0.000 | 0.000 | 0.007 | 0.094 | 0.017 | 0.000 | 0.000 |
| *IRF3* | 19 | 50162909 | 0.438 | 0.328 | 0.668 | 0.473 | 0.351 | 0.475 | 0.333 | 0.708 | 0.441 | 0.357 | 0.501 |
| *IRF3* | 19 | 50166650 | 0.017 | 0.000 | 0.067 | 0.004 | 0.000 | 0.000 | 0.001 | 0.076 | 0.006 | 0.000 | 0.000 |
| *IRF3* | 19 | 50167726 | 0.116 | 0.161 | 0.112 | 0.076 | 0.023 | 0.201 | 0.174 | 0.103 | 0.078 | 0.023 | 0.192 |
| *IRF3* | 19 | 50168926 | 0.042 | 0.000 | 0.004 | 0.001 | 0.000 | 0.000 | 0.000 | 0.000 | 0.000 | 0.000 | 0.000 |
| *IFNAR2* | 21 | 34614250 | 0.143 | 0.081 | 0.077 | 0.154 | 0.176 | 0.125 | 0.074 | 0.081 | 0.147 | 0.171 | 0.142 |
| *IFNAR2* | 21 | 34614255 | 0.351 | 0.332 | 0.190 | 0.474 | 0.582 | 0.475 | 0.333 | 0.171 | 0.448 | 0.575 | 0.522 |
| *IFNAR2* | 21 | 34634878 | 0.308 | 0.317 | 0.320 | 0.445 | 0.592 | 0.471 | 0.323 | 0.337 | 0.438 | 0.594 | 0.517 |
| *IFNAR1* | 21 | 34715699 | 0.180 | 0.138 | 0.163 | 0.252 | 0.368 | 0.261 | 0.129 | 0.163 | 0.223 | 0.377 | 0.272 |
| *IFNAR1* | 21 | 34721782 | 0.031 | 0.001 | 0.175 | 0.008 | 0.001 | 0.001 | 0.001 | 0.231 | 0.010 | 0.002 | 0.000 |
| *TMPRSS2* | 21 | 42852497 | 0.194 | 0.230 | 0.294 | 0.153 | 0.381 | 0.248 | 0.236 | 0.287 | 0.154 | 0.362 | 0.226 |
| *TMPRSS2* | 21 | 42879909 | 0.338 | 0.431 | 0.334 | 0.275 | 0.012 | 0.269 | 0.405 | 0.295 | 0.272 | 0.017 | 0.223 |
| *ACE2* | X | 15582298 | 0.012 | 0.026 | 0.003 | 0.003 | 0.000 | 0.004 | 0.018 | 0.000 | 0.004 | 0.000 | 0.001 |

**Notes:** NFE=non-Finland Europeans; AFR=sub-Saharan Africans and African Americans; AMR=admixed Americans; EAS=East Asians; SAS=South Asians; EUR=Europeans.

Supplementary Table 3. Distribution of alternative allele frequency of common variants (AAF>0.01) in the candidate gene studies according to geographic origin. Positions are based on GRCh37/hg19 assembly

| **Gene** | **Chr** | **Start** | **Campinas** | **Barretos** | **Ribeirão Preto** | **Belo Horizonte** | **ABraOM** | **BIPMed** |
| --- | --- | --- | --- | --- | --- | --- | --- | --- |
| *SLC6A20* | 3 | 45814094 | 0.103 | 0.033 | 0.054 | 0.017 | 0.048 | 0.055 |
| *SLC6A20* | 3 | 45837886 | 0.172 | 0.133 | 0.125 | 0.100 | 0.146 | 0.158 |
| *LZTFL1* | 3 | 45869972 | 0.103 | 0.117 | 0.071 | 0.083 | 0.075 | 0.082 |
| *LZTFL1* | 3 | 45875738 | 0.017 | 0.033 | 0.071 | 0.000 | 0.013 | 0.010 |
| *FYCO1* | 3 | 45960562 | 0.000 | 0.000 | 0.000 | 0.000 | 0.020 | 0.004 |
| *CXCR6* | 3 | 45987980 | 0.035 | 0.117 | 0.125 | 0.183 | 0.104 | 0.078 |
| *FYCO1* | 3 | 46007823 | 0.155 | 0.017 | 0.054 | 0.067 | 0.021 | 0.093 |
| *FYCO1* | 3 | 46007825 | 0.155 | 0.017 | 0.054 | 0.067 | 0.029 | 0.103 |
| *FYCO1* | 3 | 46007846 | 0.035 | 0.100 | 0.089 | 0.067 | 0.039 | 0.033 |
| *FYCO1* | 3 | 46008647 | 0.017 | 0.017 | 0.000 | 0.017 | 0.012 | 0.010 |
| *FYCO1* | 3 | 46008790 | 0.414 | 0.300 | 0.357 | 0.350 | 0.400 | 0.413 |
| *FYCO1* | 3 | 46009487 | 0.155 | 0.017 | 0.054 | 0.067 | 0.070 | 0.097 |
| *FYCO1* | 3 | 46009864 | 0.586 | 0.333 | 0.429 | 0.450 | 0.494 | 0.529 |
| *FYCO1* | 3 | 46009981 | 0.017 | 0.017 | 0.000 | 0.033 | 0.016 | 0.012 |
| *FYCO1* | 3 | 46010077 | 0.966 | 0.867 | 0.821 | 0.917 | 0.863 | 0.881 |
| *TLR3* | 4 | 187004074 | 0.250 | 0.267 | 0.304 | 0.183 | 0.272 | 0.336 |
| *TLR3* | 4 | 187004767 | 0.000 | 0.000 | 0.054 | 0.017 | 0.006 | 0.002 |
| *IL6* | 7 | 22771039 | 0.017 | 0.017 | 0.000 | 0.033 | 0.017 | 0.027 |
| *ABO* | 9 | 136131289 | 0.259 | 0.300 | 0.196 | 0.250 | 0.286 | 0.257 |
| *ABO* | 9 | 136131315 | 0.017 | 0.033 | 0.179 | 0.133 | 0.076 | 0.074 |
| *ABO* | 9 | 136131316 | 0.017 | 0.017 | 0.018 | 0.000 | 0.021 | 0.031 |
| *ABO* | 9 | 136131322 | 0.017 | 0.033 | 0.179 | 0.133 | 0.076 | 0.074 |
| *ABO* | 9 | 136131415 | 0.017 | 0.033 | 0.179 | 0.133 | 0.077 | 0.074 |
| *ABO* | 9 | 136131472 | 0.241 | 0.300 | 0.196 | 0.250 | 0.286 | 0.255 |
| *ABO* | 9 | 136131523 | 0.017 | 0.000 | 0.000 | 0.017 | 0.012 | 0.025 |
| *ABO* | 9 | 136131576 | 0.000 | 0.000 | 0.000 | 0.017 | 0.030 | 0.016 |
| *ABO* | 9 | 136131592 | 0.035 | 0.050 | 0.196 | 0.133 | 0.094 | 0.105 |
| *ABO* | 9 | 136131651 | 0.138 | 0.100 | 0.161 | 0.050 | 0.106 | 0.080 |
| *ABO* | 9 | 136133506 | 0.897 | 0.700 | 0.804 | 0.767 | 0.711 | 0.765 |
| *ABO* | 9 | 136135238 | 0.000 | 0.000 | 0.000 | 0.000 | 0.698 | 0.000 |
| *ABO* | 9 | 136136770 | 0.828 | 0.700 | 0.804 | 0.750 | 0.700 | 0.732 |
| *ABO* | 9 | 136136773 | 0.017 | 0.017 | 0.054 | 0.017 | 0.021 | 0.027 |
| *ABO* | 9 | 136137547 | 0.017 | 0.017 | 0.018 | 0.000 | 0.018 | 0.031 |
| *IRF7* | 11 | 613208 | 0.359 | 0.333 | 0.339 | 0.350 | 0.339 | 0.347 |
| *IRF7* | 11 | 614318 | 0.359 | 0.333 | 0.339 | 0.350 | 0.344 | 0.347 |
| *UNC93B1* | 11 | 67759316 | 0.087 | 0.135 | 0.091 | 0.173 | 0.171 | 0.056 |
| *UNC93B1* | 11 | 67763282 | 0.000 | 0.000 | 0.000 | 0.063 | 0.010 | 0.004 |
| *UNC93B1* | 11 | 67765163 | 1.000 | 1.000 | 1.000 | 1.000 | 1.000 | 1.000 |
| *STAT2* | 12 | 56737251 | 0.016 | 0.033 | 0.000 | 0.000 | 0.009 | 0.011 |
| *STAT2* | 12 | 56740682 | 0.047 | 0.067 | 0.071 | 0.017 | 0.034 | 0.033 |
| *STAT2* | 12 | 56742997 | 0.000 | 0.017 | 0.089 | 0.067 | 0.067 | 0.044 |
| *STAT2* | 12 | 56743044 | 0.031 | 0.017 | 0.018 | 0.033 | 0.026 | 0.031 |
| *TRAF3* | 14 | 103342049 | 0.281 | 0.400 | 0.411 | 0.600 | 0.385 | 0.354 |
| *FURIN* | 15 | 91419098 | 0.000 | 0.033 | 0.018 | 0.000 | 0.029 | 0.020 |
| *IRF3* | 19 | 50162909 | 0.391 | 0.417 | 0.536 | 0.400 | 0.442 | 0.443 |
| *IRF3* | 19 | 50166650 | 0.016 | 0.017 | 0.036 | 0.017 | 0.010 | 0.009 |
| *IRF3* | 19 | 50167726 | 0.094 | 0.150 | 0.125 | 0.050 | 0.141 | 0.135 |
| *IRF3* | 19 | 50168926 | 0.000 | 0.000 | 0.000 | 0.000 | 0.253 | 0.000 |
| *IFNAR2* | 21 | 34614250 | 0.156 | 0.150 | 0.214 | 0.117 | 0.103 | 0.117 |
| *IFNAR2* | 21 | 34614255 | 0.375 | 0.300 | 0.482 | 0.267 | 0.326 | 0.358 |
| *IFNAR2* | 21 | 34634878 | 0.379 | 0.233 | 0.441 | 0.214 | 0.342 | 0.237 |
| *IFNAR1* | 21 | 34715699 | 0.172 | 0.133 | 0.161 | 0.217 | 0.185 | 0.212 |
| *IFNAR1* | 21 | 34721782 | 0.031 | 0.000 | 0.036 | 0.050 | 0.039 | 0.029 |
| *TMPRSS2* | 21 | 42852497 | 0.293 | 0.200 | 0.125 | 0.133 | 0.195 | 0.206 |
| *TMPRSS2* | 21 | 42879909 | 0.276 | 0.383 | 0.304 | 0.400 | 0.355 | 0.290 |
| *ACE2* | X | 15582298 | 0.000 | 0.000 | 0.000 | 0.000 | 0.009 | 0.021 |

Supplementary Table 4. Number of peptides binders for the *HLA* alleles studied.

| **Proteins** | **Class I** | **Class II** |
| --- | --- | --- |
| ORF1ab | 35435 | 7082 |
| Orf8 | 560 | 107 |
| E | 330 | 61 |
| M | 1065 | 208 |
| N | 2050 | 405 |
| orf3a | 1330 | 261 |
| orf6 | 260 | 47 |
| orf7a | 560 | 107 |
| orf7b | 170 | 29 |
| orf10 | 145 | 24 |
| S | 6320 | 1259 |
| **Total** | **48225** | **9590** |
